# Disparities in Potentially Preventable Hospitalizations for Chronic Conditions Among Korean Americans, Hawaii, 2010–2012

**DOI:** 10.5888/pcd12.150057

**Published:** 2015-09-17

**Authors:** Hyun-Hee Heo, Tetine L. Sentell, Dongmei Li, Hyeong Jun Ahn, Jill Miyamura, Kathryn Braun

**Affiliations:** Author affiliations: Tetine L. Sentell, Dongmei Li, Kathryn Braun, Office of Public Health Studies, University of Hawaii at Manoa, Honolulu, Hawaii; Hyeong Jun Ahn, Biostatistics Core, John A. Burns School of Medicine, University of Hawaii at Manoa, Honolulu, Hawaii; Jill Miyamura, Hawaii Health Information Corporation, Honolulu, Hawaii. Dr Li is currently affiliated with the Clinical and Translational Science Institute, University of Rochester, Rochester, New York.

## Abstract

**Introduction:**

Korean Americans are a growing but understudied population group in the United States. High rates of potentially preventable hospitalizations suggest that primary care is underutilized. We compared preventable hospitalizations for chronic conditions in aggregate and for congestive heart failure (CHF) for Korean Americans and whites in Hawaii.

**Methods:**

Discharge data from 2010 to 2012 for all hospitalizations of adults in Hawaii for preventable hospitalizations in aggregate and for CHF included 4,345 among Korean Americans and 81,570 among whites. Preventable hospitalization rates for chronic conditions and CHF were calculated for Korean Americans and whites by sex and age group (18–64 y vs ≥65 y). Unadjusted rate ratios for Korean Americans were calculated relative to whites. Multivariate models, controlling for insurance type and comorbidity, provided adjusted rate ratios (aRRs).

**Results:**

Korean American women and men aged 65 or older were at greater risk of preventable hospitalization overall than white women (aRR, 2.48; *P* = .003) and white men (aRR, 1.82; *P* = .049). Korean American men aged 65 or older also were at greater risk of hospitalization for CHF relative to white men (aRR, 1.87; *P* = .04) and for older Korean American women (aRR, 1.75; *P* = .07). Younger age groups did not differ significantly.

**Conclusion:**

Older Korean American patients may have significant disparities in preventable hospitalizations, which suggests poor access to or poor quality of primary health care. Improving primary care for Korean Americans may prevent unnecessary hospitalizations, improve quality of life for Korean Americans with chronic illness, and reduce health care costs.

## Introduction

Avoidable hospitalizations is an indicator designed by the Agency for Healthcare Research and Quality (AHRQ) to be an objective measure of access to or use of high-quality primary care (1). Significant disparities are seen in preventable hospitalizations among many racial/ethnic minorities, including some Asian American subgroups, relative to their white counterparts (2–5), but preventable hospitalization rates among Korean Americans have not been reported.

Korean Americans are a growing (6) but understudied population group in the United States. Before the full implementation of the Patient Protection and Affordable Care Act (ACA), Korean Americans have had one of the highest uninsured rates among Asian Americans and tend to underutilize health services more than whites and other Asian groups (7). Of Asian American subgroups, Korean Americans have the lowest colorectal cancer screening rates, and Korean American men tend to have prostate cancer diagnosed at a later and more severe stage than do men of other racial/ethnic groups (8,9). Although the pattern of low usage of primary care is persistent in the area of preventive care and chronic disease management in Korean Americans, to our knowledge there has been no research examining preventable hospitalizations among Korean Americans.

Korean Americans have often been aggregated with “other” racial/ethnic groups when reporting rates of preventable hospitalizations, masking the potentially disproportionate rate (10). To fill the research gap, we compared the risk of preventable hospitalizations for Korean Americans and whites. We hypothesized that Korean Americans would be at greater risk of being hospitalized for preventable chronic conditions in aggregate and for congestive heart failure (CHF) compared with whites. We studied this issue in Hawaii because Hawaii has one of the highest number of foreign-born Koreans among all states (11) and the highest concentration of Korean speakers in the United States (12).

## Methods

We analyzed the Hawaii Health Information Corporation (HHIC) inpatient data (13) from January 2010 through December 2012, which were the most recent data available that included identification of Korean Americans. Data from multiple years were combined to ensure adequate numbers of preventable hospitalizations for analysis by race/ethnicity. The race/ethnicity variable is created from the race/ethnicity categories available across all hospitals. HHIC performs extensive ongoing quality assurance to confirm that racial/ethnic data are uniformly collected across all Hawaii hospitals. Only 1 primary race is reported by hospitals from patient self-report at intake, so mixed race is not available. Mixed-race individuals are represented by self-report of their primary race of identification or are included in the “other” racial/ethnic category if patients did not wish to choose 1 primary racial/ethnic identification. The discharge data also include age, sex, payer type and primary payer, and primary and secondary *International Classification of Diseases, Ninth Revision, Clinical Modification* (ICD-9) diagnosis and procedure codes.

A total of 315,635 hospitalizations for individuals aged 18 years or older were identified from January 2010 through December 2012. Korean Americans made up 1.38% of total discharges (n = 4,345), and whites made up 25.84% of total discharges (n = 81,570). We excluded pregnancies, transfers, and unknown admission sources (Korean Americans, n = 902; whites, n = 13,356) to meet AHRQ guidelines (14). Individuals not from Hawaii were also excluded to keep consistency with population totals used for rate denominators that were estimated for Hawaii residents. Under this condition, 2% of the hospitalizations were excluded among Korean Americans (n = 68), and 10% were excluded among whites (n = 6,987). After exclusions, 64,602 hospitalizations were available for analysis: 3,375 for Korean Americans and 61,227 for whites.

We used AHRQ Prevention Quality Indicators specifications version 4.5 to define 9 potential preventable hospitalizations for chronic conditions (14). More detail is available from the AHRQ website (14).

Preventable hospitalization rate denominators are typically total populations within relevant geographic areas (5,15). For the denominator for whites, we used Behavioral Risk Factor Surveillance System (BRFSS) data (16), which provide direct estimates of population size by using primary self-reported race/ethnicity, similarly to HHIC (4). Because data from multiple years provided more reliable state-level estimates, we used BRFSS from 2007 through 2010. However, BRFSS estimates for Koreans in Hawaii could not be used for several reasons. First, they exclude any foreign-born Korean Americans who do not speak English well enough to complete an English language survey. Second, the sample sizes of English-speaking Koreans in the BRFSS were extremely small and did not provide reliable estimates of the Korean Americans in Hawaii even across 4 years of data. Thus, we used 2010 US Census data, which has a Korean-language version of the questionnaire to collect information on race (17). We used the “Korean alone” group in the US Census to estimate population size in Hawaii. The “Korean-in-combination” group is not desirable for comparative analysis because individuals in this category are not mutually exclusive by race/ethnicity (6,18). We could not use US Census data for whites because the “white alone” group significantly undercounts the large percentages of mixed-race whites, particularly in a multi-ethnic state such as Hawaii (19), and because we had reliable estimates from the BRFSS.

In multivariate models, we controlled for payer (public [Medicare, Medicaid, Department of Defense], private, self-pay, and other), because it may affect rates of preventable hospitalizations. Analyses were stratified by sex and age group (18–64 y vs ≥65 y) because previous research has shown that racial/ethnic differences in rates of preventable hospitalizations vary by these demographic variables (5,20). Last, we included comorbidity that may influence preventable hospitalization rates for chronic conditions, which are likely to be case-mixed. Comorbidity was defined by the Charlson Comorbidity Index, which includes 19 categories of comorbidity that are primarily defined using ICD-9 diagnoses and procedure codes (21). Each category has an associated weight based on the adjusted risk of 1-year mortality. The total comorbidity score refers to the cumulative increased likelihood of 1-year mortality; the higher the score, the more severe the burden of comorbidity.

Characteristics of chronic preventable hospitalizations in aggregate including all 9 chronic conditions (diabetes short-term complications, diabetes long-term complications, uncontrolled diabetes without complications, diabetes with lower-extremity amputation, hypertension, CHF, angina without a cardiac procedure, chronic obstructive pulmonary disease or asthma in older adults [≥40 y], and asthma in younger adults [18–39 y]) were summarized by descriptive statistics for Korean Americans and whites. Differences in demographic variables between both groups were compared by using χ^2^ tests or Fisher exact tests for categorical variables and by using 2 independent samples *t* test or nonparametric Wilcoxon signed rank test for continuous variables. As previous work has shown (15), we used the hospital discharge as the unit of analysis to examine disparities in preventable hospitalizations between the groups. We ran sensitivity analyses using data from the first hospitalization and the last hospitalization in the analysis to ensure that disparities were not accounted for by multiple visits of individuals of both groups. Results did not differ significantly.

We calculated the unadjusted average annual rates of preventable hospitalization composite for chronic conditions for Korean Americans and whites by sex and age groups. Unadjusted rate ratios (RRs) of preventable hospitalization composite for chronic conditions were then calculated by dividing the unadjusted rates for Korean Americans by the unadjusted rate for whites. A possible disparity for Korean Americans relative to whites is represented by an RR greater than 1.0. Finally, multivariate models were developed to estimate adjusted rate ratios (aRRs) of preventable hospitalization composite for chronic conditions among Korean Americans and whites after adjusting for sex, race/ethnicity, age group, comorbidity, and insurer type. Negative binomial regression models were used to account for possible overdispersion (22). Of the 9 types of preventable hospitalizations, only CHF-related hospitalizations had a large enough sample to consider individually. Thus, a similar statistical analysis for just CHF-related hospitalizations was conducted. All data analyses were performed with SAS 9.3 (SAS Institute Inc). A 2-tailed *P* value of less than .05 was considered significant. This research was approved by the institutional review board of the University of Hawaii.

## Results

Among all nonpregnancy-related hospitalizations by any individuals aged 18 years or older per each racial/ethnic group, 3.2% of Korean Americans (n = 115) and 2.8% of whites (n = 2,014) were hospitalized for a preventable chronic condition. A total of 2,129 preventable hospitalizations were seen; Korean Americans made up 5.4% and whites 94.6%. The most frequent preventable chronic condition was CHF (Korean Americans: n = 57 [49.6%]; whites: n = 880 [43.7%]), followed by diabetes-related conditions among both Korean Americans and whites.

### Preventable hospitalizations for chronic conditions

More than half of preventable hospitalizations for chronic conditions was for patients aged 65 years or older (Table 1). Both groups differed significantly by sociodemographic characteristics such as age, age group, sex, and location of residence. However, no significant differences between Korean Americans and whites were seen by insurer type or comorbidity.

**Table 1:** Characteristics of Preventable Hospitalizations for Chronic Conditions for Korean Americans and Whites, Hawaii, January 2010–December 2012^a^

Characteristic	Korean (N = 115)	White (N = 2,014)	*P* Value
**Sex, n (%)**			
Female	63 (54.8)	833 (41.4)	.004^b^
Male	52 (45.2)	1,181 (58.6)	
**Insurance status, n (%)**			
Public	98 (85.2)	1,587 (78.8)	.24^b^
Private	14 (12.2)	380 (18.9)	
Self-pay	—c	38 (1.9)	
Other	—c	—c	
**Residence, n (%)**			
Oahu	107 (93.0)	1,178 (59.5)	<.001^b^
Neighbor islands	—c	836 (41.5)	
**Age, n (%)**			
18–64	29 (25.2)	979 (48.6)	<.001^b^
≥65	86 (74.8)	1,035 (51.4)	
**Continuous age, y, mean (SD)**	74.2 (14.2)	64.9 (17.3)	<.001 **d**
Median (range)	78 (19–98)	65 (18–104)	NA
**Charlson Comorbidity Index, mean (SD)**	4.6 (2.5)	4.2 (2.7)	.18 **d**
Median (range)	4 (0–11)	4 (0–19)	NA
**No. of hospitalizations per patient, mean (SD)**	1.2 (0.6)	1.4 (1.1)	.52 **d**
Median (range)	1 (1–4)	1 (1–17)	NA

Abbreviation: NA, not applicable; SD, standard deviation.

a Source: Agency for Healthcare Research and Quality (AHRQ), Prevention Quality Indicators version 4.5; Hawaii Health Information Corporation (HHIC) inpatient discharge databases.

b *P* values based on the χ^2^ tests (or Fisher exact tests) for categorical variables.

c This number is less than 10 and should be suppressed for privacy reasons according to the data use agreement.

d *P* values based on the 2 independent samples *t* test (or Wilcoxon signed rank test) for continuous variables.

In unadjusted models, among hospitalizations for patients aged 65 years or older, disparities in preventable hospitalizations for chronic conditions were found between Korean American women and white women (RR, 1.25); however, there was no disparity for men between groups (Table 2). Among preventable hospitalizations for patients aged 18 to 64, whites had a disparity relative to Korean Americans. However, as hypothesized, after adjusting for control variables, among hospitalizations for patients aged 65 or older, Korean American women were at greater risk for preventable hospitalization than were white women (aRR, 2.48; 95% confidence interval [CI], 1.36–4.50; *P* = .003); disparities were found between Korean American men and white men as well (aRR, 1.82; 95% CI, 1.00–3.30; *P* = .049). Among preventable hospitalizations for patients aged 18 to 64, Korean American men were more likely to be hospitalized than were white men (aRR, 1.63; 95% CI, 0.79–3.40), but the results were not significant. No disparity for women was seen between groups (aRR, 0.96; 95% CI, 0.43–2.15).

**Table 2:** Unadjusted and Adjusted Rate Ratios of Preventable Hospitalizations for Chronic Conditions for Korean Americans and Whites by Sex and Age, Hawaii, January 2010–December 2012

Age, Sex, and Race/Ethnicity	Population Totals^a^	No. of Preventable Hospitalizations for Chronic Conditions^b^	Unadjusted Annual Rate Per 100,000 Population	Unadjusted Rate Ratio	Adjusted Rate Ratio (95% CI)	*P* Value^c^
**≥65 y**						
**Female**						
Korean	3,107	51	547	1.25	2.48 (1.36‒4.50)	.003
White	33,005	433	437	1 [Reference]		
**Male**						
Korean	1,716	35	680	0.99	1.82 (1.00‒3.30)	.049
White	29,366	602	683	1 [Reference]		
**18–64 y**						
**Female**						
Korean	10,674	12	37	0.33	0.96 (0.43‒2.15)	.92
White	118,856	400	112	1 [Reference]		
**Male**						
Korean	6,040	17	94	0.66	1.63 (0.79‒3.40)	.19
White	135,936	579	142	1 [Reference]		

Abbreviation: CI, confidence interval.

a From 2010 US Census (Korean); Hawaii Department of Health Behavioral Risk Factor Surveillance System analysis (white).

b Based on Hawaii Health Information Corporation inpatient discharge data analysis.

c Based on multivariate model adjusting for age, race/ethnicity, sex, Charlson Comorbidity Index, and insurer.

### Preventable hospitalizations for congestive heart failure

Among 937 hospitalizations for CHF, 6.1% were for Korean Americans and 93.9% were for whites. Both groups differed significantly by age, age group, and location of residence. On the basis of the discharge data, Korean Americans (mean [SD], 80.8 y [10.9 y]) were older than whites (mean [SD], 73.8 y [14.0 y]) and 93% of Korean Americans lived on urban Oahu (whites, 59.5%). However, no significant differences between the groups were seen by insurer type, comorbidity, or number of hospitalizations (on average, 1 time per patient).

Among hospitalizations for patients aged 65 or older, disparities in preventable hospitalizations for CHF were found between Korean Americans and whites (women: RR, 1.13; men: RR, 1.07) (Table 3). Among preventable hospitalizations for patients aged 18 to 64, whites appear to have a disparity relative to Korean Americans in unadjusted analyses.

**Table 3:** Unadjusted and Adjusted Rate Ratios of Congestive Heart Failure (CHF) Hospitalizations for Korean Americans and Whites by Sex and Age, Hawaii, January 2010–December 2012

Age, Sex, and Race/Ethnicity	Population Totals^a^	No. of CHF Hospitalizations^b^	Unadjusted Annual Rate Per 100,000 Population	Unadjusted Rate Ratio by Population	Adjusted Rate Ratio by Population (95% CI)	*P* Value^c^
**≥65 y**						
**Female**						
Korean	3,107	29	311	1.13	1.75 (0.95‒3.23)	.07
White	33,005	272	274	1 [Reference]		
**Male**						
Korean	1716	24	466	1.07	1.87 (1.02‒3.44)	.04
White	29,366	383	434	1 [Reference]		
**18–64 y**						
**Female**						
Korean	10,674	—d	3.12	0.14	2.06 (0.51‒8.24)	.31
White	118,856	77	21	1 [Reference]		
**Male**						
Korean	6,040	—d	16	0.46	3.49 (0.36‒34.32)	.28
White	135,936	148	36	1 [Reference]		

Abbreviation: CI, confidence interval.

a From 2010 US Census (Korean); Hawaii Department of Health Behavioral Risk Factor Surveillance System analysis (white).

b Based on Hawaii Health Information Corporation inpatient discharge data analysis.

c Based on multivariate model adjusting for age, race/ethnicity, sex, Charlson Comorbidity Index, and insurer.

d This number is less than 10 and should be suppressed for privacy reasons according to the data use agreement.

After adjusting for control variables, among patients aged 65 or older, Korean American men were at greater risk of hospitalization for CHF relative to whites (aRR, 1.87; 95% CI, 1.02–3.44; *P* = .04); a similar disparity was found among Korean American women (aRR, 1.75; 95% CI, 0.95–3.23; *P* = .07). Among those aged 18 to 64, Korean Americans were more likely to be hospitalized than whites for CHF (women: aRR, 2.06; men: aRR, 3.49); however, the results for both women and men were not significant.

## Discussion

Data from Hawaii suggest significant disparities in potentially preventable hospitalizations for all chronic conditions and for CHF specifically among Korean American adults aged 65 years or older compared with whites after adjustment for comorbidity and insurer. The risk of preventable hospitalizations for chronic conditions in total among older Korean American adults was approximately 2 times that observed for white older adults. With regard to the rate of preventable hospitalizations for CHF, Korean American men also had approximately 2 times the risk of that for their white counterparts. For patients aged 18 to 64, there was a trend toward higher risk of preventable hospitalizations for chronic conditions generally among Korean American men compared with whites, and for CHF hospitalizations for both Korean American men and women; however, differences were not significant. This result may be due to the small sample size of Korean American men and women aged 18 to 64 in the multivariate model.

Despite efforts to reduce inequalities in access to high-quality primary and preventive care among racial/ethnic minority groups in the United States, health disparities still exist (2–5). Our study identifies significant disparities in preventable hospitalizations among a previously unidentified population of older Korean Americans, suggesting issues of primary health care accessibility, use, or quality in this group. It may mean older Korean Americans are less likely than older whites to have a routine source of care or to have had preventive treatments as has been seen in research comparing black and Hispanic Medicare beneficiaries with whites (3). Because Korean Americans have one of the highest uninsured rates among Asian Americans (7), the findings are not surprising. However, the findings that substantial disparities were seen among older Korean American adults relative to older white adults are particularly important, as many older Korean Americans are likely to be insured through a public payer, Medicare.

Among Korean Americans aged 18 to 64, similar pattern of higher risks of preventable hospitalizations for chronic conditions was seen relative to whites. Although statistical tests showed no significant findings, these trends suggest that effort is needed to improve use of health care for this working-age group. Compared with Korean Americans in other states, Korean Americans in Hawaii are more likely to be insured because of Hawaii’s Prepaid Health Care Act (PHCA) (23). Since 1974, PHCA requires employers to provide health care coverage to employees working at least 20 hours per week. Given the better opportunity for those of working age to be insured in Hawaii, the findings that trends toward disparities in health care persist among working-age Korean Americans are troubling.

Public programs such as Medicaid play an important role in reducing uninsured rates among racial/ethnic minority groups. Under the health insurance reform law, expansions in these programs will likely help decrease the number of uninsured Korean Americans. Beginning in 2014, 14% of Korean Americans were projected to gain Medicaid and 20% were projected to benefit from eligibility for the exchange subsidy under the implementation of the ACA (24). However, improving eligibility for access to care may not be enough to enable Korean Americans to use health care in a timely and appropriate manner. Korean Americans grapple with many challenges in accessing primary care because of language and cultural barriers, low priority and interest in preventive care, high cost, and mistrust of the US health care system (7,25). Korean American elders are mostly foreign-born with limited English proficiency and have difficulty understanding the complicated US health care system compared with the Korean system and, especially, in seeing Korean-speaking doctors or specialists in the United States. In Hawaii, some Korean Americans travel back to South Korea to seek health care with barrier-free environments (26). However, lack of usual source of care and delayed use of preventive care may put Korean Americans at risk of being hospitalized for preventable chronic conditions as they age. Limited English proficiency and lack of trust in Western medicine are related to low satisfaction with health care and delayed use of preventive screening (7). Low health literacy combined with limited English proficiency is a challenge in understanding ACA eligibility and coverage (27). Many Korean Americans with low health literacy may not be able to fully use health care even with better access after ACA implementation.

Linguistically and culturally competent outreach and enrollment workers could be good resources to connect Korean Americans to the transitional US health care system (28,29). Use of church-based interventions may also help Korean Americans use preventive care (26). Partnerships with organizations or coalitions can help monitor the performance of health care delivery and the distribution of resources related to the ACA. Action for Health Justice, a collaboration of community-based organizations and Federally Qualified Health Centers, found inaccurate translation of ACA outreach, education, and enrollment materials for Korean Americans (27). Sufficient provision of linguistically and culturally matched health professionals and well-translated education materials are needed for this hard-to-reach population. For undocumented and recently immigrated Korean Americans who are still outside of the public health safety net even under the health insurance reform, alternative coverage options should be provided. If Korean Americans would use timely and appropriate health care throughout their lifetime in affordable and comfortable health care environments, the risk of developing and being hospitalized for chronic conditions may be reduced.

### Limitations

The strength of this study is its use of population-based statewide data and a nationally recognized measure of health care performance and delivery related to chronic disease. However, administrative data has some general limitations in that it does not typically provide all possibly relevant variables, such as income or English proficiency, that may influence possible differentials in rates of preventable hospitalizations between Korean Americans and whites (30). Nationally used and validated indicators will make this research easily replicated in other settings with Korean populations. Second, different data sources (BRFSS for whites; US Census for Koreans) were used to produce each group’s denominator. We had to use different data sources because of population-level data constraints for Korean Americans. Similar data struggles have made research into Korean American populations challenging generally and help explain the paucity of research on this population in the United States. This large data gap is one of the reasons why our findings are valuable as we help to provide baseline data for a policy-relevant topic for understudied Korean American populations. Finally, because the self-reported primary race/ethnicity in HHIC data does not identify foreign-born and US-born Korean Americans, our findings must be carefully interpreted as these 2 groups may be distinct. However, heterogeneity of characteristics among Korean Americans in Hawaii with regard to nativity may be minimal compared with those in other states because Hawaii has one of the highest number of foreign-born Koreans (11) and the highest concentration of Korean-speaking populations (12) in the United States.

### Conclusions

This study is the first to investigate the risk of hospitalizations for potentially preventable chronic conditions among Korean Americans using statewide discharge data. Identifying disparities in hospitalizations for chronic conditions among Korean Americans is critical to reduce undue financial burdens and potentially to improve quality of life for this population. It may be important to develop culturally and linguistically relevant interventions to improve access to and use of health care for this population in the transitional period of health insurance reform. Future studies should focus on examining causes of the observed disparities in hospitalizations in Korean Americans by investigating differences in use of care related to cultural and cognitive barriers to care and differences in compliance with health care providers’ advice, which may be related to readmission. Investigating intrinsic but hidden barriers to health care would be important to improve the health of at-risk Korean Americans and other racial/ethnic populations in the United States.
